# Bedside Manners

**DOI:** 10.3201/eid1204.AD1204

**Published:** 2006-04

**Authors:** Christopher Wiseman

**Keywords:** Earthquake, volcano eruption, tidal wave, tsunami, epidemics

How little the dying seem to need—
A drink perhaps, a little food,
A smile, a hand to hold, medication,
A change of clothes, an unspoken
Understanding about what's happening.
You think it would be more, much more,
Something more difficult for us
To help with in this great disruption,
But perhaps it's because as the huge shape
Rears up higher and darker each hour
They are anxious that we should see it too
And try to show us with a hand-squeeze.
We panic to do more for them,
And especially when it's your father,
And his eyes are far away, and your tears
Are all down your face and clothes,
And he doesn't see them now, but smiles
Perhaps, just perhaps because you're there.
How little he needs. Just love. More Love.

**From In John Updike's Room: New and Selected Poems, by Christopher Wiseman.**

Copyright 2005 The Porcupine's Quill. Available from www.amazon.com Reprinted with author's permission.

